# Foramen ovale cannulation guided by intraoperative computed tomography with magnetic resonance image fusion plays a role in improving the long-term outcome of percutaneous radiofrequency trigeminal rhizotomy

**DOI:** 10.1007/s00701-019-03941-1

**Published:** 2019-05-17

**Authors:** Ping-Jui Tsai, Ming-Hsueh Lee, Kuo-Tai Chen, Wei-Chao Huang, Jen-Tsung Yang, Martin Hsiu-Chu Lin

**Affiliations:** 1 Department of Neurosurgery, Chang Gung Memorial Hospital, Chia-Yi Branch, 6. West Sec. Chiapu Road, Putzu City, Chia-Yi County 61636 Taiwan; 2 Chang Gung University of Science and Technology, Chia-Yi, Taiwan; 3 College of Medicine, Chang Gung University, Tao-Yuan, 33302 Taiwan

**Keywords:** Trigeminal neuralgia, Percutaneous trigeminal rhizotomy, Neuronavigation, Cerebrospinal fluid

## Abstract

**Background:**

Percutaneous radiofrequency trigeminal rhizotomy (RF-TR) is a well-established treatment for patients suffering from trigeminal neuralgia (TN) as a primary modality or for those refractory to medical treatment. However, few existing studies have identified intraoperative parameter or navigation technique that can be used to predict the rates of short-term or long-term pain relief. In this study, we analyzed patient characteristics, intraoperative parameters and technical factors, and postoperative changes in relation to immediate and long-term pain relief.

**Method:**

This study included a total 252 patients in which 340 RF-TR were performed under the guidance of intraoperative computed tomography (iCT) alone or with magnetic resonance image (MRI) and iCT fusion imaging.

**Result:**

The immediate pain relief of RF-TR with iCT alone and iCT with MR image guidance with or without cerebrospinal fluid (CSF) outflow were all above 90.4%. The 2-year pain relief rate of RF-TR using iCT alone and iCT with MR images guidance with or without CSF outflow were 47.8%, 39.8%, 71.7%, and 53.9% respectively. Significant factors for 2-year pain relief were CSF outflow, iCT with MR image fusion, non-recurrent TN, and presence of postoperative facial numbness.

**Conclusion:**

This preliminary study demonstrated foramen ovale cannulation under the aid of iCT with MR image guidance could improve 2-year pain relief.

## Introduction

Percutaneous radiofrequency trigeminal rhizotomy (RF-TR) is a well-established and effective technique for treating trigeminal neuralgia (TN) [25]. The short-term pain relief of RF-TR is good, which has a reported immediate and 2-year pain relief rates of 80–99% and 40–85% respectively; and when TN recur, most do within 2 years after the initial RF-TR [14, 16, 24, 26, 32, 33, 37, 39, 41]. Accurate positioning of the rhizotomy needle in a timely manner is essential to ensure patient comfort and to prevent inadvertent puncturing of nearby neurovascular structures causing serious complications such as carotid-cavernous fistula, cranial nerve injuries, intracranial hemorrhage, and internal carotid artery injury [1, 12, 15]. Efforts to improve long-term treatment success of RF-TR have been made, but only few factors have been identified. Favorable prognostic factors are classical TN [7, 16, 22, 29, 40], higher ablation temperature of up to 68 °C [27, 36], accurate site of thermal lesioning, and postoperative hypoesthesia [32].

RF-TR is usually done under the guidance of fluoroscopy; however, computed tomographic or navigation assistance can be employed for added safety and precision, especially for the less experienced surgeon. RF-TR with intraoperative computed tomography (iCT) navigation became the treatment of choice in our center since August 2010, which progressed further to include fusion images of computed tomography and magnetic resonance image (MRI); this progression was based on the fact that prior image guidance techniques only allowed the accurate cannulation of the foramen ovale, whereas the inclusion of MRI enabled visualization of the trigeminal cistern and ganglion, which we hypothesize could facilitate better anatomical localization of the target for lesioning. We have shown that this technique can shorten the procedure time and is easy to master, and although short-term pain relief is demonstrated, the long-term advantage of the added image assistance remains unknown [6, 17]. In this study, we assessed the efficacy of RF-TR under the guidance of MRI and iCT fusion imaging and explored factors that are associated with long-term outcome and complications.

## Methods

### Patient population

Two hundred fifty-two consecutive patients, who were diagnosed with medically refractory TN and received RF-TR under iCT navigation at Chiayi Chang Gung Memorial Hospital, were enrolled from May 2010 to February 2016. Two hundred thirty patients had classical TN; 18 had tumor with nerve compression, and 4 had post-herpetic neuralgia. Of the patients, 162 (64.3%) were women and 90 (35.7%) were men. The patient’s age ranged from 20 to 90 years (mean, 62.8 ± 11.3 years). Other clinical characteristics are shown in Table 1. These patients underwent a total of 340 RF-TN procedures. Two patients who had bilateral TN received bilateral RF-TR, and 68 patients had multiple RF-TR. iCT navigation with MRI fusion was used in 131 patients, while iCT navigation without MRI fusion was used in 121 patients.

**Table 1:** Patient characteristics (*n* = 252)

Patient characteristics	
Age (years)	62.8 ± 11.3 (20–90)
Gender (*n*)	
Male	90
Female	162
Symptoms distribution (*n*)	
V1 only	0
V2 only	48
V3 only	62
V1 + 2	12
V2 + 3	116
V1 + 2 + 3	14
Side of pain (*n*)	
Right	155
Left	95
Bilateral	2
Etiology (*n*)	
Classic	230
Tumor	18
Post-herpes neuralgia	4
Times of RF-TR (*n*)	
1	184
2	51
3	14
4	3
Previous non-medical treatment (*n*)^×^	
Microvascular decompression	10
RF-TR	88
Balloon compression	1
Radiosurgery	32

*V1*, ophthalmic nerve; *V2*, maxillary nerve; *V3*, mandibular nerve

^×^Some patients received more than one kind of non-medical treatment

### Procedure

All procedures were performed on an outpatient basis at our Brain-SUITE® iCT. The MRI series were taken at the outpatient department before the procedure, and the CT images were acquired intraoperatively before cannulation of the foramen ovale. The trigeminal cistern was marked on all axes of the MRI slices, and the foramen ovale and the lateral pterygoid plate were highlighted on all axial slices of CT images. The MRI were fused with the CT images on the iPlan® 2.0 platform with Cranial Essential & Unlimited® 1.0 software (Brainlab, Germany).

**Fig. 1:**
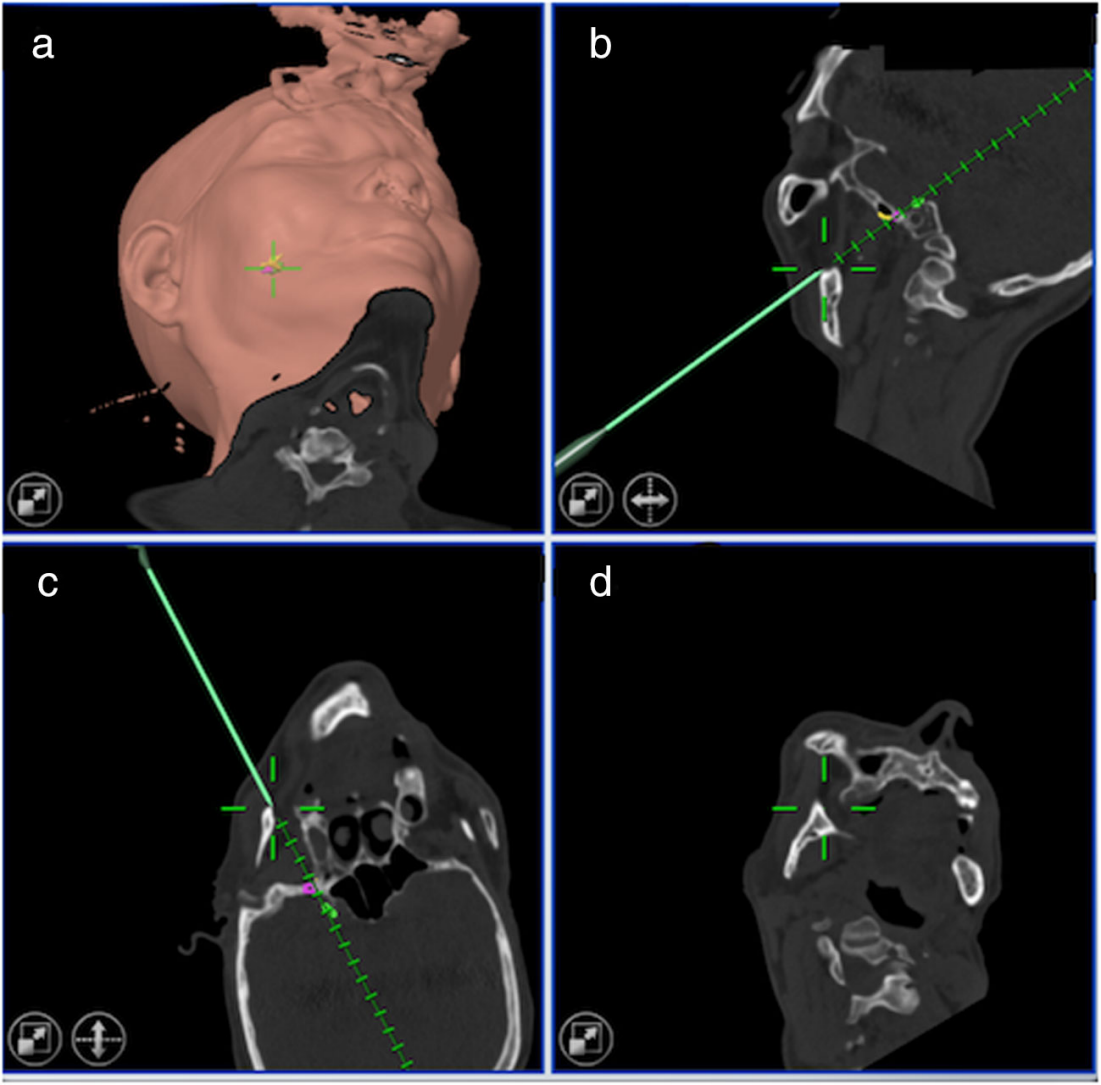
The intraoperative images of a 68-year-old man who underwent RF-TR by the use of the intraoperative CT with MRI fusion-guided technique. The trajectory (green line) was chosen along an unobstructed linear pathway to penetrate foramen ovale (purple circle) into the trigeminal cistern (green circle). The lateral pterygoid plate (yellow line) should be avoided during penetration. Three-dimentional skin probe eye view (**a**), inline sagittal view (**b**), inline axial view (**c**), and probe eye view (**d**)

**Fig. 2:**
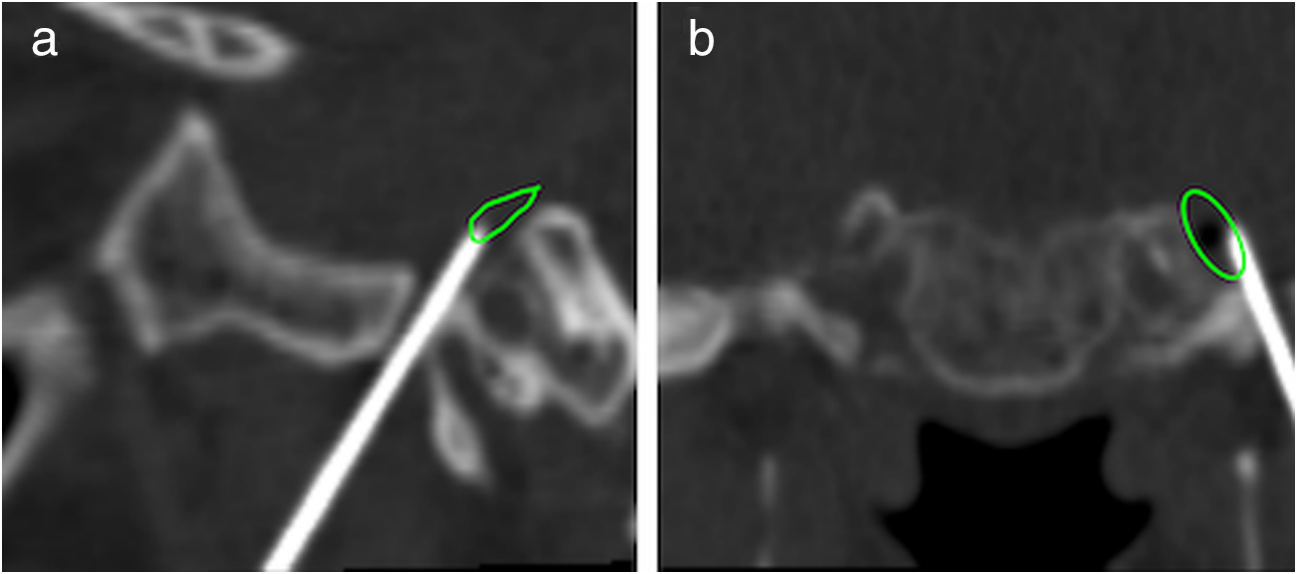
Computed tomography scan confirms the position of needle is adequate. Inline sagittal view (**a**) and inline coronary view (**b**)

The patient was placed in supine position with their head in slight extension on a snug-fitting horseshoe headrest. The hub of the needle was attached to the instrument adaptor, and the foramen ovale was cannulated based on the Hartel’s technique with adjustments by fusion image guidance. The target of the needle tip was 6 mm beyond the endocranial surface of foramen ovale based on the CT image guidance without MRI fusion; by contrast, the needle tip was placed into the trigeminal cistern based on MRI fusion guidance (Fig. 1). The location of the needle tip differed depending on the affected distribution of TN. The needle tip was shallower and more lateral in the trigeminal cistern for V3 distribution, and deeper for V1 distribution. The iCT scan was repeated to confirm the location of needle tip with MRI fusion (Fig. 2). A Tew electrode kit and a Radionics RTG-3CF generator were used (Radionics, Burlington, MA, USA). Stimulation test given by 50 Hz, 1 ms, and 0–1 V until paresthesia in the distribution of symptoms was done before two consecutive lesions were made at a temperature of 60–95° for 100 s. The intraoperative parameters such as stimulating voltage, ablation temperature, and CSF outflow were recorded. All intraoperative parameters are listed in Tables 2 and 3.

**Table 2:** Procedural characteristics of the navigation method groups (*n* = 340)

	iCT alone	iCT with MRI guidance	*p* value
Procedure numbers (*n*)	189	151	
Patient characteristics Age (years)	62.15 ± 11.01	63.86 ± 11.52	0.77
Gender (male:female) Symptoms distribution (*n*)	70:119	52:99	0.46
V2 only	43 (22.8%)	19 (12.6%)	0.02
V3 only	41 (21.7%)	35 (23.2%)	0.11
V1+2 V2+3	6 (3.2%) 84 (44.4%)	9 (5.9%) 82 (54.3%)	0.21 0.07
V1+2+3	15 (7.9%)	6 (4.0%)	0.13
NRS before RF-TR	9.59 ± 0.62	9.42 ± 0.81	0.13
Recurrent TN^+^ (*n)*	49 (25.9%)	60 (39.7%)	< 0.01
Intraoperative parameters			
Stimulating voltage (V)	0.18 ± 0.11	0.22 ± 0.11	0.87
Temperature (°C)	69.03 ± 5.59	75.46 ± 6.10	0.50
CSF outflow (*n*)	23 (12.2%)	99 (65.6%)	< 0.01
Postoperative change			
NRS after RF-TR	2.17 ± 3.40	0.88 ± 1.92	0.01
Facial numbness^√^	29 (15.3%)	79 (52.3%)	< 0.01
Masseter weakness	30 (15.9%)	19 (12.6%)	0.74
Duration of pain-relief			
No improvement	18 (9.5%)	4 (2.6%)	< 0.01
Less than 2 years	94 (49.7%)	48 (31.8%)	< 0.01
More than 2 years	77 (40.8%)	99 (65.6%)	< 0.01

^+^Non-recurrent TN means patients had received non-medical treatment before

^√^Facial numbness included dysthesia and hypoesthesia

**Table 3:** Procedural characteristics of the outcome groups (*n* = 340)

	No difference after procedure	Recurrence within 2 years	Pain-relief for more than 2 years	Total
Procedure number (*n*) Patients characteristics	22 (6.5%)	142 (41.7%)	176 (51.8%)	340
NRS before RF-TR	9.90 ± 0.29	9.52 ± 0.60	9.45 ± 0.81	9.51 ± 0.71
Non-recurrent TN (*n*)	19 (8.3%)	86 (37.2%)	126 (54.5%)	231
Recurrent TN (n)	3 (2.8%)	56 (51.3%)	50 (45.9%)	109
Intraoperative parameters				
Stimulating voltage (V)	0.18 ± 0.14	0.20 ± 0.11	0.20 ± 0.11	0.20 ± 0.11
Temperature (°C)	70.7 ± 7.6	71.4 ± 6.9	72.3 ± 6.12	71.8 ± 6.58
iCT alone (*n*)	18 (9.5%)	94 (49.7%)	77 (40.8%)	189
With CSF outflow	2 (8.7%)	10 (43.5%)	11 (47.8%)	23
Without CSF outflow	16 (9.6%)	84 (50.6%)	66 (39.8%)	166
iCT/MRI fusion (*n*)	4 (2.6%)	48 (31.8%)	99 (65.6%)	151
With CSF outflow	1 (1.0%)	27 (27.3%)	71 (71.7%)	99
Without CSF outflow	3 (5.7%)	21 (40.4%)	28 (53.9%)	52
Postoperative change				
Facial numbness	1 (0.9%)	26 (24.1%)	81 (75%)	108
NRS after RF-TR	9.90 ± 0.29	1.81 ± 2.53	0.41 ± 1.13	1.57 ± 2.87

### Data collection

Two hundred fifty-two patients were followed up for 2–8 years via subsequent outpatient visits or a phone interview. A numeric rating scale (NRS) score was used to evaluate the severity of pain before and after the procedure. The NRS, the interval to recurrence and facial numbness (included different degrees of hypoesthesia and dysthesia), as well as the occurrence of postoperative complications such as anesthesia dolorosa, diplopia, keratitis, and masseter weakness were recorded during serial follow-up.

### Statistics

The patients were grouped according to the type of image guidance used (iCT alone versus iCT with MRI fusion); the patient and procedural characteristics were compared using the Mann-Whitney *U* test and chi-square test. The clinical outcome in relation to ablation temperature, CSF outflow, and complications (odds ratios [ORs]) were analyzed by univariate binary logistic regression analysis. All factors with a *p* value < 0.2 by univariate analysis were considered in the multivariate model using a backward elimination strategy. A *p* value < 0.05 was considered statistically significance. Missing data were censored in all analyses. All data analyses were performed with the Statistical Product and Service Solutions (SPSS) version 23.0.

## Results

### Patient characteristics and operative findings

Comparison of the patient groups using iCT alone and iCT with MRI fusion is shown in Table 2; the two groups had comparable age, gender, and preoperative NRS; however, significantly, more patients with recurrent TN and fewer isolated maxillary nerve involvement were seen in the group using iCT with MRI fusion. While the simulating voltage and ablation temperature were similar between the two groups, iCT with MRI fusion was associated with a significantly increased occurrence of CSF outflow from 11.6 to 65.6%, a greater degree of immediate pain relief from a mean NRS of 2.17 ± 3.40 to 0.88 ± 1.92, and a higher likelihood of a durable response lasting more than 2 years from 40.8 to 65.6%.

### Outcome and risk factors

The procedure characteristics according to various outcome groups are listed in Table 3; in a total of 340 procedures, 6.5% did not respond to treatment; 41.7% had recurrence within a 2-year period, and the remaining 51.8% were pain-free 2 years after the procedure. iCT alone and iCT with MRI guidance resulted in an immediate pain relief rate of 90.5% and 97% respectively, and a corresponding rate of 40.8% and 65.6% for pain relief lasting more than 2 years.

On univariate analysis of the patient characteristics, intraoperative parameters, and postoperative changes, immediate pain relief was related to CSF outflow, iCT with MRI guidance and postoperative facial numbness, and negatively correlated to neuralgia involving the V1 dermatome; in a multivariate model, postoperative facial numbness and involvement of V1 distribution remained statistically significant (Table 4). The failure rate in the 36 patients who had V1 involvement was 13.9%.

**Table 4:** Risk factors of immediate pain relief

Factors	OR (95% CI)	*p* value
Univariate analyses		
Patients’ characteristics		
Older age	0.99 (0.95–1.03)	0.66
Male Gender	0.66 (0.25–1.69)	0.37
Involved V1 distribution	0.34 (0.12–0.99)	0.048
Involved V2 distribution	0.30 (0.07–1.35)	0.12
Involved V3 distribution	0.50 (0.15–1.75)	0.28
Non-recurrent TN	1.27 (0.49–3.23)	0.62
Intraoperative parameters		
Stimulating voltage	7.71 (0.09–657.87)	0.37
Ablation temperature	1.01 (0.94–1.08)	0.83
CSF outflow	3.84 (1.11–13.25)	0.03
iCT/MRI fusion	3.87 (1.28–11.69)	0.02
Postoperative change		
Facial numbness	7.66 (1.02–57.83)	0.048
Multivariate analyses		
Facial numbness	13.86 (1.58–121.64)	0.02
Involved V1 distribution	0.14 (0.04–0.50)	0.03

Significant factors for pain relief lasting more than 2 years on univariate analysis were younger age, CSF outflow, iCT with MRI guidance, non-recurrent TN, postoperative numbness, and involvement of the V2 dermatome. CSF outflow, iCT with MRI guidance, non-recurrent TN, and postoperative facial numbness remained statistically significant in a multivariate model (Table 5).

**Table 5:** Risk factors of 2-year pain relief

Factors	OR (95% CI)	*p* value
Univariate analyses		
Patients’ characteristics		
Older age	0.98 (0.96–0.99)	0.044
Male Gender	0.73 (0.46–1.21)	0.18
Involved V1 distribution	1.59 (0.74–3.43)	0.24
Involved V2 distribution	2.24 (1.30–3.84)	< 0.01
Involved V3 distribution	0.68 (0.41–1.14)	0.14
Intraoperative parameters		
Stimulating voltage	2.50 (0.35–17.92)	0.36
Ablation temperature	0.98 (0.95–1.16)	0.30
CSF outflow	3.04 (1.87–3.93)	< 0.01
iCT/MRI fusion	2.53 (1.59–3.98)	< 0.01
Postoperative change		
Facial numbness	2.42 (1.02–57.83)	0.048
Multivariate analyses		
Facial numbness	2.10 (1.17–3.79)	0.01
Non-recurrent TN	2.38 (1.28–3.85)	< 0.01
CSF outflow	1.84 (1.003–3.38)	0.048
iCT/MRI fusion	1.97 (1.12–3.46)	0.02

The immediate pain relief rate following repeated RF-TR for patients suffering from recurrent TN was 97.5%, which was comparable to non-recurrent cases receiving their first RF-TR; however, the rate of recurrence in this patient group was 43.6% at 2 years. On univariate analysis, significant factors for 2-year pain relief in the group of recurrent TN were younger age, involvement of V2 distribution, CSF outflow, iCT with MRI guidance, and postoperative facial numbness; however, no factors remained statistically significant in a multivariate model (Table 6).

**Table 6:** Risk factors of 2-year pain relief in recurrent TN

Factors	OR (95% CI)	*p* value
Univariate analyses		
Patients’ characteristics		
Older age	0.96 (0.93–0.99)	0.02
Male gender	0.63 (0.28–1.40)	0.25
Involved V1 distribution	3.19 (0.83–12.35)	0.09
Involved V2 distribution	3.08 (1.26–7.53)	0.01
Involved V3 distribution	1.06 (0.45–2.47)	0.90
Intraoperative parameters		
Stimulating voltage	6.21 (0.27–141.24)	0.25
Ablation temperature	0.97 (0.91–1.03)	0.26
CSF outflow	5.12 (2.21–11.86)	< 0.01
iCT/MRI fusion	2.28 (1.02–5.08)	0.04
Postoperative change		
Facial numbness	2.51 (1.04–6.05)	0.04
Multivariate analyses		
No factors had statistical significance		

### Complication

One hundred eight patients (31.8%) had postoperative facial numbness after RF-TR; the percentage of postoperative facial numbness was lowest in patients that had no pain relief after RF-TR (4.5%) and highest in those that had a durable response for more than 2 years (46%). There were 13 patients (3.8%) who complained of severe dysesthesia and anesthesia dolorosa developed in 6 patients (1.8%). Masseter weakness was observed in 49 (14.4%) of 340 procedures and these patients reported no disability, and the weakness improved gradually. No patient suffered from oral cavity penetration, diplopia, corneal keratitis, CSF leakage, carotid-cavernous fistula, oculomotor nerve palsy, or abducens nerve palsy. The complications had no correlation with the ablation temperature, CSF outflow, or previous treatment modality.

## Discussion

Neuronavigation is a widely adopted technology in modern neurosurgery [3, 4, 6, 9, 11, 17, 18, 27, 28, 35, 36]. The trajectory for RF-TR is chosen along an unobstructed linear pathway through the foramen ovale, and the placement of needle tip usually does not exceed the clival line confirmed by fluoroscopy [4, 11, 18]. In this study, the entry point, trajectory and position of the needle tip, was guided by neuronavigation with further needle adjustments as necessary based on the electrophysiological response. The immediate and 2-year pain relief rates were comparable with other studies; CSF outflow, iCT with MRI fusion, non-recurrent TN, and postoperative facial numbness were favorable prognostic factors.

The occurrence of CSF outflow during cannulation was sixfold higher when MRI fusion was used compared to iCT alone, which was also associated with a better 2-year outcome. This can be due to the fact that CSF may act as an effective conductive medium for heat transfer by convection around the gasserian ganglion within the trigeminal cistern, and that CSF may prevent tissue charring around the needle tip for an effective lesioning volume [20, 21]. While CSF outflow can be observed when the tip of the cannula is inside the trigeminal cistern, which is taken as an important confirmatory sign during RF-TR with iCT and MRI fusion, it can also occur outside of the trigeminal cistern such as the cerebellopontine angle cistern or mesio-temporal cistern, in which the absence of CSF may indicate the needle tip is inside the rootlets of the trigeminal nerve, thus, is predictive of good outcome [5, 14, 31].

The common complications after RF-TR are severe dysesthesia (3.7–25%), anesthesia dolorosa (0.5–2%), and masseter weakness (4.1–30%). The less common complications are keratitis (0.4–4%), diplopia (0.2–4%, most transient), CSF leak (0.16%), meningitis (0.2%), carotid- cavernous fistula (0.06–0.14%), and blindness [1, 12–15, 19, 32, 34]. Our study displayed a similar profile; these common complications are unavoidable because these are the consequences of gasserian ganglion lesioning; other than that, no other complications occurred in our study. The ablation temperature for treatment of TN represents a balance between achieving maximal pain relief while minimizing facial numbness and painful dysesthesia; an optimal ablation temperature of 75 °C is recommended by Tang et al., which is correlated to an effective pain control with the lowest incidence of painful dysesthesia, but should be guided by the voltage required during motor and sensory test stimulation [30]. There was no correlation between ablation temperature and complication in our study, which ranged from 60 to 90 °C; this could be due to the precise localization of the electrode inside the trigeminal cistern. Postoperative facial numbness was a bothering and unavoidable state following successful thermal lesioning in our study; this finding is consistent with other reports [5, 31]. The position of needle should be in the gasserian ganglion or in the triangular plexus [31], which measures approximately 5.8–6.3 mm from the foramen ovale in cadaver studies [2, 15]; the use of MRI fusion enabled direct anatomical localization of the gasserian ganglion, with a safe needle penetration of up to 9.24 mm beyond the endocranial margin of the foramen ovale compared to 4.06 mm using iCT neuronavigation alone [6], this technique effectively eliminated all puncture-related complications in our study. While ablation temperature is correlated with lesioning complications, this was not found in our study probably because of the accurate positioning of the needle.

Treatment of TN with isolated or concomitant V1 involvement by RF-TR is controversial [13, 38]; the reported recurrence rate in this patient group can be as high as 30–60% [10]; therefore, microvascular decompression should remain as an option in the treatment algorithm [25]. TN with V1 involvement was also identified as a negative predictor for good outcome in our study, in that as many as 13.9% of patient in this group failed to obtain immediate pain control. This may be related to our conservatism towards treating TN with V1 component in order to preserve normal corneal sensation. Anatomically, V1 rootlets is most difficult to reach owing to its distant and superior location in the needle trajectory; this division can be better targeted using a curved needle with lesioning performed under careful observation for the diminution of the direct and consensual blink reflex [10]. Method to improve outcome of TN with V1 involvement includes the addition of pulsed radiofrequency after continuous radiofrequency thermocoagulation; this technique has been shown to reduce recurrence and the incidence and recovery time of corneal hypoesthesia [38].

Recurrent TN was found to be a poor prognostic factor in subsequent RF-TR, as more than half suffered from relapsing pain in spite of initial pain relief following repeated RF-TR; however, outcome predictors for RF-TR in patients with recurrent TN could not be identified. The pathophysiological mechanism of recurrent TN is not well understood; it could be related to sparing of the trigger myelinated A-beta fibers by radiofrequency rhizotomy, which selectively destroys A-delta and C fibers [8], and the persistence of peripheral pathogenic mechanism leading to reformation or reactivation of the central algogenic focus [23], thus is unlikely to be resolved by technical factors described in our study.

The voltage threshold for pain reproduction on test stimulation was not predictive of the outcome, whereas other technical factors such as the use of iCT guidance with MRI fusion and the occurrence of CSF outflow were favorable prognostic factors, thus a shift in needle targeting from electrophysiological-based stimulation to image-based anatomical localization could have the potential to eliminate the ambiguity in pain reproduction of test stimulation, and enables a greater degree of patient comfort from a deeper level of sedation during the procedure.

## Conclusion

The present study demonstrated that accurate anatomical localization by the use of the iCT with MRI fusion could avoid all puncture-related complications to result in a good outcome at 2 years, and CSF outflow using this technique is a favorable predictor for long-term pain relief.

## Abbreviations

CSF: Cerebrospinal fluid
iCT: Intraoperative computed tomography
MRI: Magnetic resonance image
NRS: Numeric rating scale
ORs: Odds ratios
RF-TR: Percutaneous radiofrequency trigeminal rhizotomy
TN: Trigeminal neuralgia
V1: Ophthalmic nerve
V2: Maxillary nerve
V3: Mandibular nerve
